# Supplementary material for: SCRMshaw: Supervised cis-regulatory module prediction for insect genomes
Source: PLoS One. 2024 Dec 5;19(12):e0311752. doi: 10.1371/journal.pone.0311752 (PMC11620701; doi:10.1371/journal.pone.0311752)
Supplement: S1 File — (PDF) [file pone.0311752.s001.pdf]

Jul 04, 2024 Version 2

SCRMshaw: supervised cis-regulatory module prediction for insect genomes V.2

DOI
dx.doi.org/10.17504/protocols.io.e6nvw1129lmk/v2

Hasiba Asma[1], Luna Liu[2], Marc S. Halfon[1,2,3,4]

[1]Program in Genetics, Genomics, and Bioinformatics; [2]Department of Biomedical Informatics;
[3]Department of Biochemsitry; [4]Department of Biological Sciences , University at Buffalo-State University of New York

Halfon Lab

Marc S. Halfon
University at Buffalo-State University of New York

OPEN ACCESS

DOI: dx.doi.org/10.17504/protocols.io.e6nvw1129lmk/v2

Protocol Citation: Hasiba Asma, Luna Liu, Marc S. Halfon 2024. SCRMshaw: supervised cis-regulatory module prediction for insect genomes. **protocols.io** https://dx.doi.org/10.17504/protocols.io.e6nvw1129lmk/v2Version created by **Marc S. Halfon**

License: This is an open access protocol distributed under the terms of the **Creative Commons Attribution License**, which permits unrestricted use, distribution, and reproduction in any medium, provided the original author and source are credited

Protocol status: Working
We use this protocol and it's working

Created: May 22, 2024

Last Modified: July 04, 2024

Protocol Integer ID: 102839

Keywords: regulatory genomics; enhancers; insects; Drosophila; cis-regulatory module; genome annotation

Funders Acknowledgement:
USDA
Grant ID: 2019-67013-29354

Abstract

As the number of sequenced insect genomes continues to grow, there is a pressing need for rapid and accurate annotation of their regulatory component. SCRMshaw is a computational tool designed to predict *cis*-regulatory modules (CRMs) in the genomes of various insect species (Kantorovitz 2009; Kazemian 2011; Asma and Halfon 2019; Asma 2024). One of the key advantages of SCRMshaw is its accessibility. It requires minimal resources—just a genome sequence and training data from known *Drosophila* CRMs, which are readily available for download. Even users with modest computational skills can run SCRMshaw on a desktop computer for basic applications, although an HPC cluster is recommended for optimal results. SCRMshaw can be tailored to specific needs. Users can employ a single set of training data to predict CRMs associated with a particular gene expression pattern, or utilize multiple sets to provide a rough regulatory annotation for a newly-sequenced genome.

This protocol provides an update to the protocol in Kazemian and Halfon (2019) and incorporates modifications introduced in Asma and Halfon (2019) and Asma et al. (2024).

Guidelines

Computational considerations:
The SCRMshaw_HD pipeline can analyze an average-sized insect genome using several training sets in a matter of hours; we are able to run all but the largest or most fragmented genomes with our full default set of 48 training sets in under 72 hours. The bulk of the computational time is spent in the SCRMshaw step. To decrease run times, chromosomes and/or training sets can be split out and run as separate instances on additional sets of 25 nodes, if available, as a simple parallelization strategy. Storage space increases with genome size, mostly due to the larger number of kmers that must be stored, and can grow to several TB with larger genomes. However, the majority of this space can be released upon completion of the pipeline by deleting intermediate and temporary files, using “cleanup” scripts we have made available. If sufficient temporary storage space is not available, it is advisable to run the original lightweight SCRMshaw (Kazemian and Halfon, 2019) rather than SCRMshaw_HD, which will keep storage requirements below 100GB for most genomes.
Running *Orthologer* using Singularity:
Orthologer is currently distributed as a Docker container. Many HPC environments do not allow deployment of Docker files, but can run Singularity (Aptainer) images. To run *Orthologer* using Singularity, you must first create a Singularity image from the *Orthologer* Docker image. You may need to do this on a local machine and then transfer the Singularity image to your HPC cluster.

From the Ortholger GitLab site (https://gitlab.com/ezlab/orthologer_container):

## Note on singularity

The latest docker2singularity (v4.1.0) seems to produce errors when converting the docker image (error relocating /usr/local/singularity/bin/singularity: ...).
It has been tested successfully on v3.9.9.

The command used to convert orthologer:3.2.2 to a singularity image:
```
docker run -v /var/run/docker.sock:/var/run/docker.sock -v /tmp/test:/output --privileged -t --rm
quay.io/singularity/docker2singularity:v3.9.9 orthologer:3.2.2
```

The output singularity file will be created at `/tmp/test/orthologer_3.2.2-.sif .*

To run the image one can e.g do:
```
singularity shell /tmp/test/orthologer_3.2.2-<date and hash>.sif
```
and then at the prompt type `run_tests`.

In the current directory, this will create a directory `test` from where all tests are run.
##
On our cluster, we find these additional changes are also necessary:

(1) Replace the command “docker run” with “singularity exec”
(2) Omit the “-u $(id -u)” flag
(3) Substitute “-H” for “-v” in all of the *Orthologer* commands, e.g. “-H $(pwd):/obdwork”
(4) Make sure to substitute the Singularity .sif image for the Docker image in all commands, e.g. “/orthologer_3.2.2-<date and hash>.sif” instead of “/ezlabgva/orthologer:v3.2.2”

So for example, the command at protocol step 28 would be:

```
singularity exec -H $(pwd):/odbwork /path/to/ezlabgva_orthologer_v3.2.2-<date and hash>.sif orthologer -c create
```

Making these changes as necessary, steps 28-37 can otherwise be followed as provided.

Safety warnings

If using an HPC cluster, it is essential to work with your system administrator to make sure the necessary software is correctly installed. Docker is often not available on HPC platforms; see the section “Initializing Orthologer” for recommendations on running this component.

Before start

Software and Dependencies:

Ensure that the following software is installed :

- **Python:**

Python3

Pybedtools

Statistics

Pandas

Numpy

Biopython (for “extract_unannoatatedScaffolds.py”)

- **Perl:**

Perl

Bioperl

- **Other:**

MACS2

Download - **https://pypi.org/project/MACS2/**

NCBI Download Utility (optional)

Download - **https://www.ncbi.nlm.nih.gov/home/tools/**

- **Optional (for Orthology assignment steps):**

Docker

Download - **https://www.docker.com/products/docker-desktop/**

Orthologer Software

Requirements: Docker on Mac or Linux

Download - https://hub.docker.com/r/ezlabgva/orthologer (ver2.4.3)

Documentation - https://hub.docker.com/r/ezlabgva/orthologer

- **Optional (for final merging of the SCRMshaw results)**

BEDTools (https://github.com/arq5x/bedtools2/releases)

Create Project directory

1 Create a Project Directory and subdirectories

Command

"create project directory"

```
mkdir project1
```

2 Under the project directory create subdirectories for saving input and output files respectively, for training data, and for scripts

Command

"create subdirectories"

```
mkdir GenomeFiles HD_output TrainingSets Scripts
```

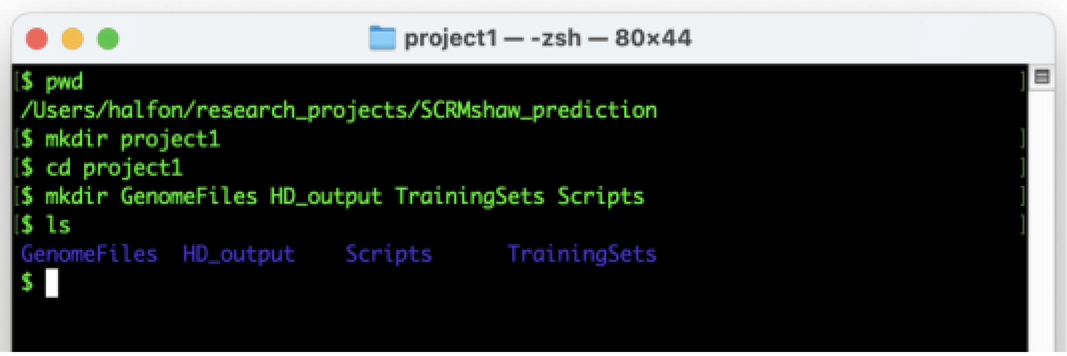

Figure 1: Sample commands to create project directory

Note

Note: If using SCRMshaw in a multi-user setting, or if planning to analyze many genomes, other directory structures might be more practical. For example, the “Scripts” and “TrainingSets” directories can be placed outside of the “project” directory where they will be accessible for multiple projects. Should you choose a different directory structure, be sure to update paths in the code examples below as necessary.

Install SCRMshaw

3 From the project directory, clone the SCRMshaw_HD software from https://github.com/HalfonLab .

Command

“Download SCRMshaw”

```
git clone https://github.com/HalfonLab/SCRMshaw_HD.git
```

Note

See note above about using a different directory structure for multi-user or multi-project settings.

4 If necessary, decompress the tar archive (e.g., tar xvf SCRMshaw_HD.tar). This will create a single “SCRMshaw_HD” directory with the following subdirectories:

```
bin/
src/
code/
include/
example/
```

5 Navigate to the SCRMshaw directory

Command

“Navigating to the SCRMshaw directory"

```
cd SCRMshaw_HD
```

6 Run “make.” Detailed information on how to install and run the software is provided in the “README.txt” file located within the software package.

Command

“Executing the “make” command”

```
make
```

Obtain training set sequences

7 Obtain up-to-date training data from https://github.com/HalfonLab/Training_sets.
Find details on how to construct custom training sets in Kazemian and Halfon (2019). Place training sets into the “TrainingSets” directory.

Download required genome files

8 Download the genome files from NCBI Datasets (https://www.ncbi.nlm.nih.gov/datasets/) and place them in the GenomeFiles directory.
Necessary files are:

- Genome sequences (FASTA)
- Annotation features (GFF)
- Protein (FASTA)

We recommend using the RefSeq builds if available.

Note

If planning to run SCRMshaw on multiple genomes, we recommend making subdirectories for each genome, e.g., project1/GenomeFiles/SpeciesX. In the code examples that follow, make sure to update paths to reflect the directory structure you create.

Note

Even if not running SCRMshaw on the *Drosophila melanogaster* genome, the *D. melanogaster* protein FASTA file is required for the Orthology step. Obtain this from https://www.ncbi.nlm.nih.gov/datasets/taxonomy/7227/.

Note

Download files using the NCBI download utility (**Software Tools - Download - NCBI**). Find a sample script to facilitate on our GitHub page at: https://github.com/HalfonLab/UtilityPrograms/blob/master/getGenomes.sh.

Note

The file "assembly_data_report.jsonl" contains metadata about the assembly and release and store for future reference. Use the NCBI "dataformat" tool to convert this to more readable formats. Discard other files accompanying the download.

9 Rename the "genomic.gff" and "protein.faa" files to "SpeciesX.gff" and "SpeciesX_protein.fs", respectively (where "X" is the name of your species). This avoids future confusion due to the generic "genome" and "protein" designations, and provides compatibility for downstream steps.

Command

"Rename the "genomic.gff"

```
mv genomic.gff SpeciesX.gff
```

Command

"Rename the "protein.faa"

```
mv protein.faa SpeciesX.fs
```

The GenomeFiles directory should now look similar to this:

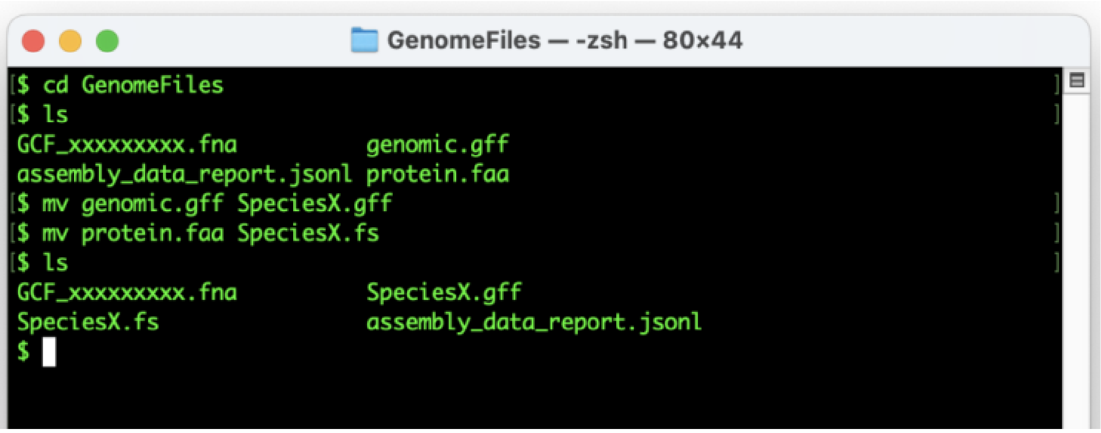

Figure 2: Example contents of GenomeFiles directory

Assessing genome files using preflight

10 Assess genome and annotation files for proper content and formatting using our preflight script, which can be downloaded from https://github.com/HalfonLab/UtilityPrograms. We recommend placing this into the "Scripts" directory.

Command

"running preflight"

```
perl ../Scripts/preflight.pl -gff genome.gff -fasta GCF_xxxxxxxxx.fna
```

10.1 Preflight validates the formats of these files and produces a comprehensive log file that highlights any issues along with basic information such as the number of chromosomes/scaffolds and their sizes, data types present in the annotation (e.g., ‘gene’, ‘exon’, ‘ncRNA’, etc.), and average intergenic distances.
Preflight also provides a sample output of the SCRMshaw-generated ‘gene’ and ‘exon’ files. This feature allows users to identify any discrepancies or errors stemming from the input files and to reformat these files as needed before running SCRMshaw. Be sure to check the following:

- In the first section, no GFF3 errors are reported
- Gene and Exon data appear in the following sections
- The ‘FASTA’ section reports “all sequences have proper characters”
- The following section reports that “All seqids in GFF are also in FASTA”

If any of these conditions is not met, correct the input files before moving on to run SCRMshaw, or SCRMshaw will fail.

Removing unannotated genes

11 Preflight will also indicate whether there are sequence scaffolds that do not contain any annotated genes (reported as “seqids not in the GFF file.”) Any such sequence scaffolds should be removed before passing the genome sequence to the main SCRMshaw program. Perform this using the “extract_unannotatedScaffolds.py” script (from https://github.com/HalfonLab/UtilityPrograms) that takes the preflight output file and FASTA sequence file as input and generates a fasta file, “mappedOnly_GCF_xxxxx.fna”, that contains only the desired sequences.

Command

“extracting unannotated scaffolds”

```
python ../Scripts/extract_unannotatedScaffolds.py -po
preflight_output_file.txt -fasta GCF_xxxxxxxxx.fna
```

Note

The extract_unannoatatedScaffolds.py script requires biopython

Masking Tandem Repeats

12

Note

Tandem repeats, as the name indicates, consist of a repeated pattern of one or more nucleotides that occur directly after each other. An example would be ACACACACAC, where the dinucleotide "AC" is repeated five times. The occurrence of a long tandem repeat in the training dataset or the region to be scored significantly skews the distribution of k-mer counts toward the repeated pattern, which would result in assignment of a "false" high score to regions with one or more occurrences of the repeated pattern. To avoid this potential issue, tandem repeats in both the training datasets and the genome to be searched are masked prior to running SCRMshaw using Tandem Repeat Finder (Benson, 1999). To mask tandem repeats:

Download the current version of TRF for your computer architecture from https://tandem.bu.edu/trf/home (e.g., trf409.linux64).

Command

"example download command for TRF"

```
curl -JLO ../Scripts/ https://github.com/Benson-Genomics-Lab/TRF/releases/download/v4.09.1/trf409.linux64
```

13 Change the downloaded TRF file to an executable form:

Command

"changing to executable"

```
chmod +x ../Scripts/trf409.linux64
```

14 Run TRF on your FASTA files (e.g., mappedOnly_GCF_xxxxx.fna) using parameters as shown:

Command

“running TRF”

```
../Scripts/trf409.linux64 mappedOnly_GCF_xxxxx.fna 2 7 7 80 10 50 500
-m -h
```

- The resulting output file will be named

“mappedOnly_GCF_xxxxx.fna.2.7.7.80.10.50.500.mask”

Note

- Different genomes have different repeat content and character. We are currently investigating whether to adjust the TRF parameters on a per-genome basis. However, we have found these parameters to be generally effective for a wide range of genomes.
- TRF substitutes the repeated nucleotides with “N” characters that can be fed directly into SCRMshaw. If using a different tool for masking repeats, be sure that the repeated nucleotides are replaced by the same number of “N” characters.
- If you have constructed custom training sets, make sure to also run TRF on the positive and negative training sequences, as described in Kazemian and Halfon (2019).

Running SCRMshaw

15 SCRMshaw requires the following files and directories:

1. A genome file (e.g., “genome.fa”), a multi-FASTA file including all of the genomic sequences needing to be scored. This is typically a downloaded genome file that has been masked for tandem repeats, and is passed to the program using the “--genome" option.
2. One or more dataset directories each containing two files, “crms.fasta” and “neg.fasta.” These files are respectively the positive and negative training sequences, both in FASTA file format. We highly recommend tandem repeat masking of genome.fa, crms.fasta, and neg.fasta.
3. A text file containing a list of the dataset directories (e.g., “trainingSet.lst”; see below step 17). This is specified using the “--traindirlst” option.

16 Navigate back to the project directory.

Command

"move back to the project directory"

```
cd ..
```

17 Under the project directory, create a list file "trainingSet.lst" that contains the path to the training datasets you wish to use (e.g., project1/TrainingSets/dataset1/), one line per dataset. You can create this file using a simple text editor or using the following "echo" commands (adjust path as necessary):

Command

"creating file for dataset1"

```
echo "~/project1/TrainingSets/dataset1/" >> trainingSet.lst
```

Command

"creating file for dataset2"

```
echo "~/project1/TrainingSets/dataset2/" >> trainingSet.lst
```

Command

“creating file for dataset3”

```
echo "~/project1/TrainingSets/dataset3/" >> trainingSet.lst
```

Note

If using SCRMshaw in a multi-user setting and the “TrainingSets” directory is located outside the project directory (as discussed in the Note at Step 2) then adjust the paths in “trainingSet.lst” accordingly (e.g., “~/TrainingSets/dataset1”).

At the conclusion of these steps, your directory should resemble Figure 3 below.

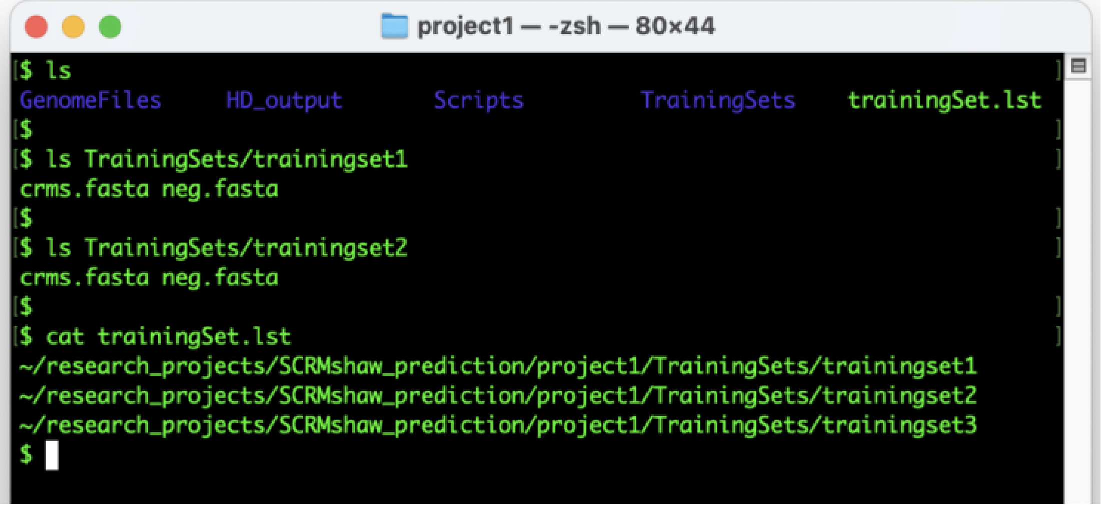

Figure 3: SCRMshaw Training Set Sample

18 Run SCRMshaw by providing the full paths to the genome file, gene annotation file, and training set list after the --genome, --gff, and --traindirlst flags, respectively (e.g. GenomeFiles/SpeciesX.gff), and providing appropriate values for --lb and --outdir (see Note).

Command

“Running SCRMshaw”

```
perl SCRMshaw_HD/code/scrm.pl --thitw 5000 --gff
GenomeFiles/SpeciesX.gff --
genomeGenomeFiles/mappedOnly_GCF_xxxxx.fna.2.7.7.80.10.50.500.mask --
traindirlst trainingSet.lst --imm --
```

18.1 We recommend that the base directory for --outdir be set to ~/project1/HD_output for consistency with the examples in this protocol.

Note

- SCRMshaw_HD requires running 25 concurrent instances with the “window start” incremented by 10 for each instance.
- For optimal efficiency, we recommend performing this computation on a computing cluster. Execute each of the 25 instances independently, enabling straightforward parallelization.
- An illustrative script utilizing the Slurm manager is available for download as "Slurm_script_SCRMshawHD.sh" from GitHub (https://github.com/HalfonLab/UtilityPrograms/blob/master/Slurm_script_SCRMshawHD.sh); this script sets appropriate values for the $myNUM and $SLURM_TASK_DIR variables in the code example above.
- If a different workflow manager, other than Slurm, is employed, ensure the correct syntax for the respective scheduler and appropriate values for the --lb and --outdir parameters. File names also need to be generated correctly to prevent any disruptions to downstream scripts.
- This process is analogous to merely adjusting the default shift size parameter from 250 bp to 10 bp and adhering to the fundamental SCRMshaw protocol (as described in Kazemian and Halfon, 2019), allowing for execution on a single processor. However, the latter approach would substantially increase the execution time, especially for a large genome.

Note

To execute traditional SCRMshaw (not the "HD" version), set the --lb option to 0, and specify an appropriate file name for "--outdir". If using Slurm, remove the sbatch --array option (e.g. "#SBATCH --array=1-25") from the Slurm script.

19 The output of SCRMshaw for the 25 offset instances will be saved in subdirectories, such as 'task_offset_0_1', 'task_offset_10_2', 'task_offset_20_3', and so on up to 'task_offset_240_25', as specified by the '--outdir' parameter in the Slurm script. These directories will be created

automatically if they don't already exist. If Slurm is not utilized, it is crucial to ensure that the output directory and file names adhere to the specified format (e.g., 'task_offset_0_1' to 'task_offset_240_25') to prevent any downstream disruptions.

20 The SCRMshaw command line options include:

20.1 Use the name of scoring method(s) for prediction ("--imm" for IMM, "--hexmcd" for HexMCD, and "--pac" for PAC): required. Select any individual or combination of methods to run (e.g. "--imm --pac" to run IMM and PAC). For details about the individual scoring methods see (Kantorovitz et al. 2009, Kazemian et al. 2011).

20.2 The gene annotation file of the genomic regions to be scored in GFF3 format (e.g., "--gff SpeciesX.gff"): optional but strongly recommended.

- Alternatively, separate "gene" and "exon" files can be used. It is also possible to use SCRMshaw without providing any annotation, but this will not allow for exclusion of coding sequences or calculation of "local rank" (see below).

Note

- Individual "gene" and "exon" files can be used in lieu of an annotation GFF3 file; one or both files can be provided.
- These should be tab-delimited lists of gene or exon coordinates, respectively, in the form chromosome (or scaffold), start coordinate, end coordinate, strand (+ or -), gene ID/exon ID (e.g., gene_name:1).
- In the absence of an annotation GFF3 file, running SCRMshaw without a "gene" file will preclude calculation of local rank and provision of results indicating closest flanking genes.
- Omitting an "exon" file will prevent masking of exons and allow coding regions to be scored as potential CRMs, which is strongly discouraged.

20.3 The number of top scoring regions to be reported as CRM predictions (--thitw N): optional, default = 2000.

Note

- Adjust the reported number of predictions depending on expectations of how many CRMs might be recovered, for instance based on how functionally broad or specific the training CRMs are.
- At the moment, we do not have a systematic way of learning this parameter from the input training sets. We recommend using a generous value, and then if desired working with a smaller subset of results obtained using the "Generate_top_N_SCRMhits" script that can be downloaded from https://github.com/HalfonLab/UtilityPrograms/blob/master/Generate_top_N_SCRMhits.pl

21 The offset parameter --lb: optional, default = 0. The --lb tells the algorithm to ignore the base pairs at the beginning of a chromosome/scaffold before this point, i.e., to start creating the

analysis windows from this point in the genome. The Slurm script described above sets --lb from 0 to 240 with a step size of 10 (i.e 10, 20, 30,..., 240) for the 25 instances being run for each training set.

22 Which steps of the SCRMshaw pipeline to run (--step 123): optional, default = 123. Step 1 processes the genome and genome annotation, step 2 processes the training data, and step 3 scores the genome.

Note

- For a given genome, we recommend first running SCRMshaw with a limited number of training datasets and the "--step" option set to "--step 123."
- This will process the genome and annotation, and ensure that create all of the proper subdirectories.
- Parallel instances of SCRMshaw can then be run with additional training sets and "--step 23." To run an additional scoring method for a previously run training set (e.g., if only "--imm" was run initially and you now want to add "--hexmcd"), only "--step 3" is necessary.

23 Detailed information on how to run SCRMshaw from the command line and additional available options can be obtained by the following command: "perl SCRMshaw_HD/code/scrm.pl" or from the README document accompanying the SCRMshaw distribution.

- An example benchmark data set along with how to run and interpret its results is provided in the example directory within the software package.

Post-processing

24 The post-processing steps merge the data from the 25 SCRMshaw instances and select the top-scoring regions as CRM predictions. To run the post-processing steps, download the following scripts from GitHub and place in the
"Scripts" directory:

24.1 post_processing_complete.sh (https://github.com/HalfonLab/UtilityPrograms)

24.2 Generate_top_N_SCRMhits.pl (https://github.com/HalfonLab/UtilityPrograms)
postProcessingScrmshawPipeline.py
(https://github.com/HalfonLab/post_processing_SCRMshaw_pipeline)

24.3 Execute the shell script post_processing_complete.sh from the project directory. Use the path to the GFF annotation file for SCRMshaw and enter as a command line argument:

Command

“post-processing”

```
./Scripts/post_processing_complete.sh GenomeFiles/SpeciesX.gff
```

24.4 The post-processing procedure will create the following files: scrmshawOutput_offset_0to240.bed: this is the concatenated results of the top 5000 results from each of the individual SCRMshaw instances

24.5 scrmshawOutput_peaksCalled_[hexmcd/imm/pac]: BED-formatted files with the MACS-generated peaks with the results for each training set and SCRMshaw method

24.6 peaks_AllSets.bed: the concatenated final results from the three separate peaks files in BED format

24.7 log_flankingMoreThanOneGenesFromAllSets.txt: a log file documenting cases of SCRMshaw predictions where there is more than a single flanking gene either up- or downstream of the prediction (e.g., where genes overlap or are nested).

Note

Make sure the following dependencies are available for this step:

- Pybedtools
- Statistics
- Pandas
- numpy
- MACS2

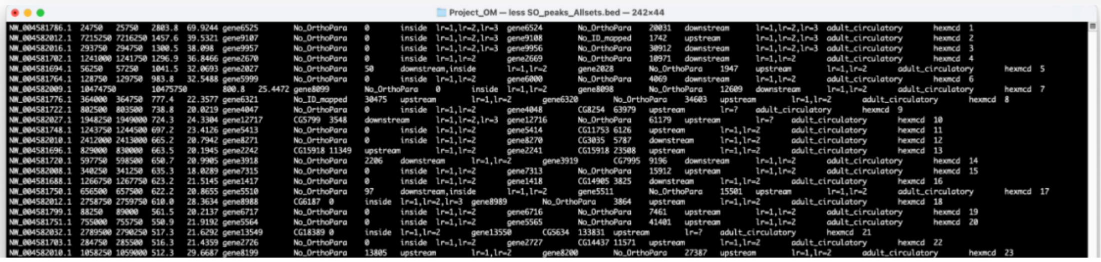

Figure 4: Final output from the post-processing script

Note

This file can also be used as an input for pcrmeval.py (Asma and Halfon, 2019) for evaluation of individual training sets.

24.8 It is possible to finish your SCRMshaw processing at this step, and use the information from the peaks_AllSets.bed file for any desired downstream analysis.

- However, we recommend continuing with the Orthology Mapping steps below, as it makes the output more understandable by providing recognizable gene names to what otherwise would be arbitrary ID numbers.
- If you choose to end your SCRMshaw processing here, we recommend you still follow the “cleanup” procedures in the final section of this protocol, “Cleaning Up.”

Note

The “peaks_AllSets.bed” may have duplicate and/or overlapping predictions, e.g. when the same or similar sequences are predicted by more than one training set, or more than one method. This can easily be resolved using BEDTools (Quinlan and Hall, 2010) as described below in the “Interpreting the Output” section.

Running Orthologer

25

Note

This step maps genes listed in the SCRMshaw output file to their orthologs in *Drosophila melanogaster* using Orthologer (Kuznetsov et al., 2023), a program developed by the Zdobnov lab to map orthologs. This is helpful, although not necessary, for making use of SCRMshaw output.

Note

Orthologer is currently distributed as a Docker container. Many HPC environments do not allow deployment of Docker files. It may be simpler to execute the following steps locally on a desktop Mac or Linux computer. Alternatively, you may be able to run the Docker image as a Singularity (Aptainer) image; consult with your HPC system administrator. (See “Guidelines” for tips on running *Orthologer* using Singularity.)

Before running Orthologer, create a directory titled Project_OM.

Command

"Make Project_OM directory for running Orthologer"

```
mkdir Project_OM
```

26 Navigate to the Project_OM directory

Command

"Navigate to the Project_OM directory"

```
cd Project_OM
```

Note

This directory needs to be completely empty or the *Orthologer* setup won't work.

27 Obtain the Orthologer Docker image (see note above about Docker and HPC platforms).

Command

"Pulling Orthologer from Docker"

```
docker pull ezlabgva/orthologer:v3.2.2
```

Note

This example shows us using version 3.2.2 of Orthologer. However, you may use the latest stable version instead. Remember to update the version numbers in subsequent steps if you use a different version. Check the release notes for any changes that might need to be made in the commands to run the program.

28 Set a working environment in directory $(pwd) as user $(id -u):

Command

"Set Orthologer working environment"

```
docker run -u $(id -u) -v $(pwd):/odbwork ezlabgva/orthologer:v3.2.2
orthologer -c create
```

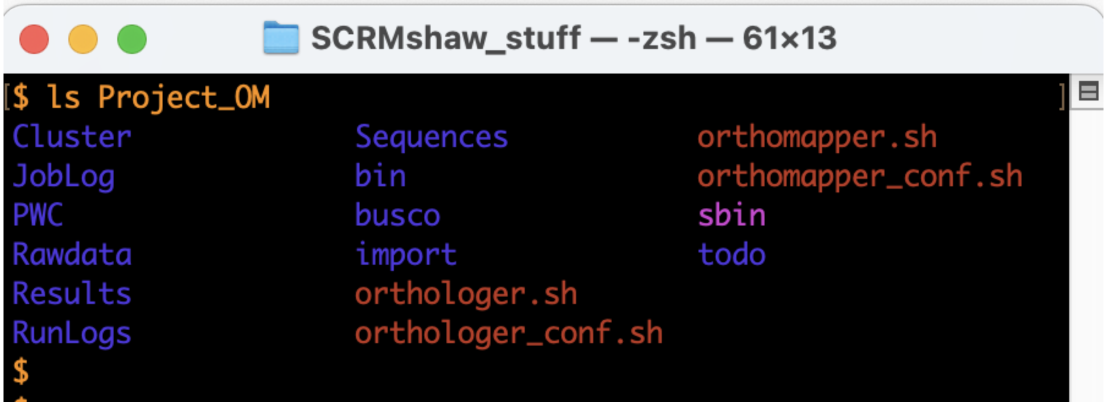

Figure 5: Orthologer starting files after pulling from Docker

29 Create a subdirectory of Project_OM titled "protein_files".

Command

“Create protein_files directory”

```
mkdir Project_OM/protein_files
```

30 Copy the protein FASTA files for the current species as well as for *Drosophila melanogaster* into the protein_files directory.

Command

“Copy protein FASTA files”

```
cp GenomeFiles/SpeciesX_protein.fs path/DMEL_protein.fs
Project_OM/protein_files
```

Note

Check that the file extensions for the protein FASTA files is “.fs”. If necessary, change the extension.

31 Create a text file specifying the FASTA files to be used, as follows:

Command

“Create ‘mydata.txt’ file”

```
for x in $(ls files/*.fs); do echo "+$(basename $x .fs) $x"; done >
mydata.txt
```

32 Import the specified proteomes:

Command

"import proteomes"

```
docker run -u $(id -u) -v $(pwd):/odbwork ezlabgva/orthologer:v3.2.2
./orthologer.sh manage -f mydata.txt
```

33 Run the Orthologer container

Command

"Running Orthologer"

```
docker run -u $(id -u) -v $(pwd):/odbwork ezlabgva/orthologer:v3.2.2
./orthologer.sh -t todo/mydata.todo - r all
```

Saving Orthologer outputs for orthology mapping

34 Note

Orthologer creates output files in the Results, Cluster and Rawdata directories. Our outputs need to be saved into a directory titled "orthologer_output" to use for orthology mapping. The user should retain three files: "DMEL_protein.fs.maptxt", "SpeciesX_protein.fs.maptxt", and the "mydata.og_map" file:

- Cluster/mydata.og_map: this file has the list of all internally mapped IDs (ortho/paralogs) between *Drosophila melanogaster* and species X
- Rawdata/DMEL_protein.fs.maptxt, Rawdata/SpeciesX_protein.fs.maptxt: these are the mapping files of internal gene_IDs to IDs in the original protein FASTA files

The remaining output files can be discarded

Navigate back to the main project directory

Command

“Navigate back to the main project directory”

```
cd ..
```

35 Create a directory “orthologer_output”

Command

“create “orthologer_output” directory

```
mkdir orthologer_output
```

36 Copy the desired Orthologer output files to the orthologer_output directory

Command

“copy files”

```
cp Project_OM/Cluster/mydata.og_map
Project_OM/Rawdata/SpeciesX_protein.fs.maptxt
Project_OM/Rawdata/DMEL_protein.fs.maptxt orthologer_output/
```

37 If desired, at this point the Project_OM directory and its remaining files can be deleted.

Command

“removing the Project_OM directory”

```
rm -r Project_OM/
```

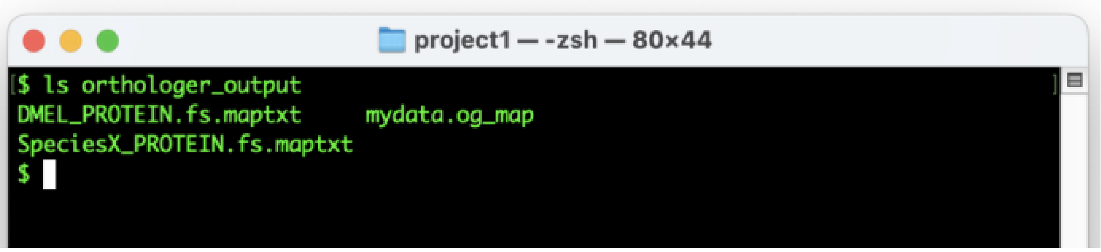

Figure 6: Output files from Orthologer required for Orthology Mapping

Mapping DMEL orthologs to SpeciesX SCRMshaw predictions

38

Note

The previous steps map SpeciesX proteins to their *D. melanogaster* orthologs, but our SCRMshaw predictions have associated gene IDs and not protein IDs. We therefore need an intermediate mapping file to associate protein IDs and gene IDs. This mapping file can be generated based on information in the SpeciesXannotation GFF file. However, there is considerable variation in how the necessary information is presented in GFFs compiled by different research groups. Therefore, you may need to customize your approach depending on the exact nature of the annotations.

The below line of code will work when the relevant gene ID and protein ID are both listed for a CDS annotation line, with the correct gene ID given as “gene”.

Generate a gene ID ⇔ protein ID mapping file

Command

“Sample code for generating a gene⇔protein mapping file (Using Perl)”

```
perl -F'\t' -ane 'next if $_ =~ /#/; if ($F[2] =~ /CDS/){$F[8] =~
/gene=(.*?);.*protein_id=(.*)/; print "$1\t$2\n";}' speciesX.gff |
sort -u > speciesX_A.map
```

Command

“Sample code for generating a gene⇔protein mapping file (Using sed)”

```
sed -n '/#/d; /CDS/ {s/.*gene=\([^;]*\);.*protein_id=\
([^;]*\).*/\1\t\2/; p; }' speciesX.gff | sort -u > speciesX_A.map
```

Note

Because there are often multiple CDS lines for a single gene, we piped the above command through “sort -u” to remove any duplicate lines. We recommend this step of removing duplicate lines, either at this step or following final editing of the mapping file, before running the “OM_mappingFlyOrthologsToSCRMshawPredictions.py” script.

39 Inspect the speciesX_A.map file and check that the first column of this file has the gene IDs formatted the same as that in column 6 of the SCRMshaw output file, and that the protein IDs in the second column match those in the Orthologer-generated “mydata.og_map” file. If necessary, edit these files for appropriate formatting. The following figures provide an example:

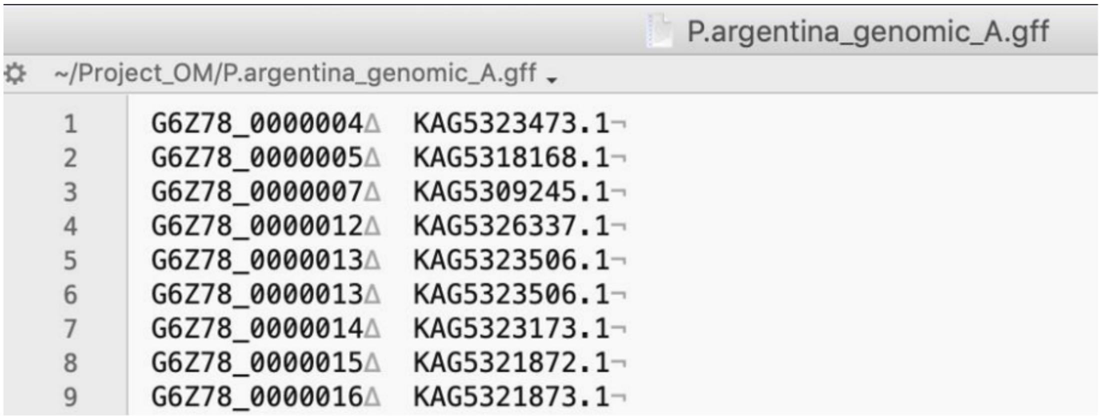
P.argentina_genomic_A.gff
~/Project_OM/P.argentina_genomic_A.gff

```
G6Z78_0000004	KAG5323473.1
G6Z78_0000005	KAG5318168.1
G6Z78_0000007	KAG5309245.1
G6Z78_0000012	KAG5326337.1
G6Z78_0000013	KAG5323506.1
G6Z78_0000013	KAG5323506.1
G6Z78_0000014	KAG5323173.1
G6Z78_0000015	KAG5321872.1
G6Z78_0000016	KAG5321873.1
```

Figure 7: P.argentina_A.map without editing

39.1 Looking at this file we can see that protein ID (2nd) column matches the format of the Orthologer output, but the gene ID (1st) column is formatted slightly differently than SCRMshaw output (the latter have ‘gene-’ at the beginning of each ID). We therefore edit the file as follows:

Command

“editing P.argentina_A.map”

```
sed 's/^/gene-/' P.argentina_A.map > P.argentina_Aedited.map
```

39.2 The resulting file should look like this:

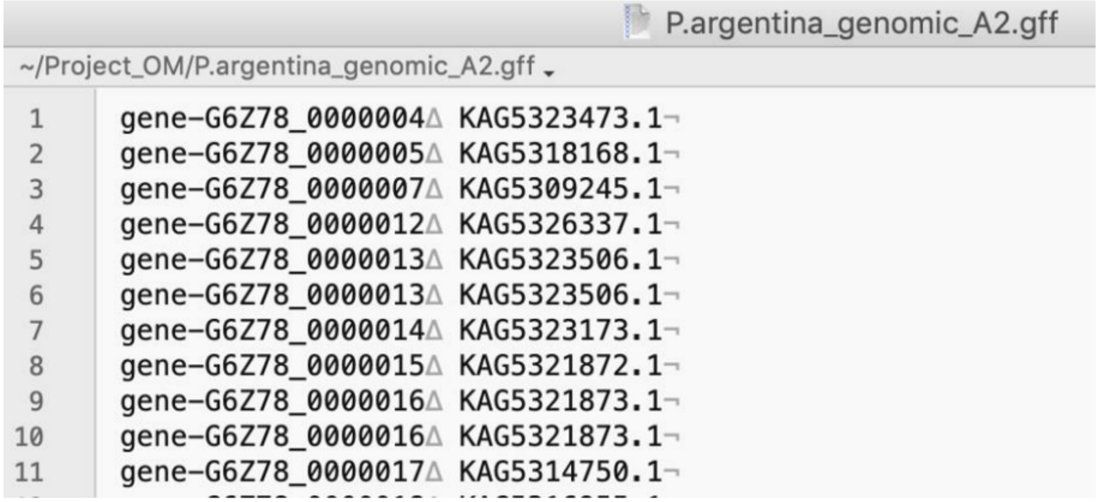

P.argentina_genomic_A2.gff
~/Project_OM/P.argentina_genomic_A2.gff

```
gene-G6Z78_0000004	KAG5323473.1
gene-G6Z78_0000005	KAG5318168.1
gene-G6Z78_0000007	KAG5309245.1
gene-G6Z78_0000012	KAG5326337.1
gene-G6Z78_0000013	KAG5323506.1
gene-G6Z78_0000013	KAG5323506.1
gene-G6Z78_0000014	KAG5323173.1
gene-G6Z78_0000015	KAG5321872.1
gene-G6Z78_0000016	KAG5321873.1
gene-G6Z78_0000016	KAG5321873.1
gene-G6Z78_0000017	KAG5314750.1
```

Figure 8: P.argentina_Aedited.map

40 Obtain the necessary scripts and files from the Halfon Lab GitHub

Command

"Clone the GitHub repository"

```
git clone https://github.com/HalfonLab/Mapping-D.mel-Orthologs.git
```

41 Run the script "OM_mappingFlyOrthologsToSCRMshawPredictions.py".
You will need to supply the following command line options:

- –ft: A table of *D. melanogaster* annotation features (included in the Mapping-D.mel-Orthologs repository)
- –mD: *D. melanogaster* Orthologer "maptxt" output file (included in the Mapping-D.mel-Orthologs repository or from your output in the Orthologer step)
- –mX: The SpeciesX Orthologer "maptxt" file generated in the Orthologer step
- –og: The "mydata.og_map Orthologer output file generated in the Orthologer step
- –sp1id: The appropriately edited SpeciesX.map file from step 40
- –so: The SCRMshaw final output "peaks_Allsets.bed" file

Command

command title = “Running the final orthology mapping”

```
python Mapping-D.mel
Orthologs/OM_mappingFlyOrthologsToSCRMshawPredictions.py -ft Mapping-
D.mel
Orthologs/GCF_000001215.4_Release_6_plus_ISO1_MT_feature_table.txt -
mD Mapping-D.mel-Orthologs/DMEL_PROTEIN.fs.maptxt -mX
orthologer_output/SpeciesX_protein.fs.maptxt -og
orthologer_output/mydata.og_map -sp1id SpeciesX_Aedited.map -so
peaks_Allsets.bed
```

Interpreting the output

42 The output file will have the same file name as SCRMshaw’s output file with an additional “SO_” prefix. As noted above, this file may have duplicate and/or overlapping predictions, e.g. predict the same or similar sequences by more than one training set, or more than one method. This can be resolved using the “sort” and “merge” commands in BEDTools (Quinlan and Hall, 2010) as follows:

Command

“BEDTools Sort and Merge”

```
bedtools sort -i SO_peaks_Allsets.bed | bedtools merge -c
4,5,6,7,8,9,10,11,12,13,14,15,16,17,18 -o
max,max,distinct,distinct,distinct,distinct,distinct,distinct,distinct
,distinct,distinctdistinct,distinct,distinct,min
```

Note

The output file is an 18-column tab delimited file as follows:

- Chromosome
- start
- end
- Peak amplitude
- SCRMshaw score
- flanking gene
- *D. melanogaster* ortholog of flanking gene
- distance of hit from flanking gene (basepairs)
- location of hit relative to flanking gene
- local rank
- next closest flanking gene
- *D. melanogaste*r ortholog of next flanking gene
- distance of hit from flanking gene (basepairs)
- location of hit relative to flanking gene
- local rank
- training set
- method (hexmcd, imm, pac)
- rank

If the orthologous gene is not known, it will be listed as “No_OrthoPara.” Where predictions were merged, multiple results may be provided in each column, depending on the results of the merge (e.g., for method, “imm, hexmcd”). Peak amplitude, score, and rank will contain the best value from among the merged predictions.

Note

This approach was designed to be minimally restrictive in that it does not enforce a one-to-one ortholog mapping. In cases of likely paralogs, we consider all of the paralogs as a potential result.

Cleaning up

43

Note

SCRMshaw creates a large number of files when processing the genome and training data, which contain breakdowns of individual chromosome/scaffold FASTA sequences (/fasta/chr), details on k-mer frequencies (/fasta/kmers), and
FASTA sequences of each 500 bp window used in scoring (/fasta/windows). For a large genome, these files can require significant storage space, but for routine applications are not required once the predicted CRM results have been obtained.
We provide a simple utility shell script, “cleanup_fastaAndScoredirectory_SCRMshawHD.sh,” that will delete the contents of the /fasta and /scores directories to free up storage space.

Download the “cleanup_fastaAndScoredirectory_SCRMshawHD.sh” from (https://github.com/HalfonLab/UtilityPrograms) and place into the “Scripts” directory.

44 From the Project1 directory, run the cleanup script.

Command

“cleanup directories”

```
./Scripts/cleanup_fastaAndScoredirectory_SCRMshawHD.sh
```

Protocol references

1. Asma, H., and Halfon, M.S. (2019). Computational enhancer prediction: evaluation and improvements. BMC Bioinformatics 20, 174.

2. Asma, H., Tieke, E., Deem, K.D., Rahmat, J., Dong, T., Huang, X., Tomoyasu, Y., and Halfon, M.S. (2024). Regulatory genome annotation of 33 insect species. bioRxiv, 2024.2001.2023.576926.

3. Benson, G. (1999). Tandem repeats finder: a program to analyze DNA sequences. Nucleic Acids Res 27, 573-580.

4. Kantorovitz, M.R., Kazemian, M., Kinston, S., Miranda-Saavedra, D., Zhu, Q., Robinson, G.E., Gottgens, B., Halfon, M.S., and Sinha, S. (2009). Motif-blind, genome-wide discovery of *cis*-regulatory modules in *Drosophila* and *mouse*. Dev Cell 17, 568-579.

5. Kazemian, M., and Halfon, M.S. (2019). CRM Discovery Beyond Model Insects. Methods Mol Biol 1858, 117-139.

6. Kazemian, M., Zhu, Q., Halfon, M.S., and Sinha, S. (2011). Improved accuracy of supervised CRM discovery with interpolated Markov models and cross-species comparison. Nucleic Acids Res 39, 9463-9472.

7. Kuznetsov, D., Tegenfeldt, F., Manni, M., Seppey, M., Berkeley, M., Kriventseva, E.V., and Zdobnov, E.M. (2023). OrthoDB v11: annotation of orthologs in the widest sampling of organismal diversity. Nucleic Acids Res 51, D445-D451.

8. Quinlan, A.R., and Hall, I.M. (2010). BEDTools: a flexible suite of utilities for comparing genomic features. Bioinformatics 26, 841-842
