# Supplementary material for: SCRMshaw: Supervised cis-regulatory module prediction for insect genomes
Source: PLoS One. 2024 Dec 5;19(12):e0311752. doi: 10.1371/journal.pone.0311752 (PMC11620701; doi:10.1371/journal.pone.0311752)
Supplement: S2 File — (PDF) [file pone.0311752.s002.pdf]

Validation report for genomic_Amel_Hav3.1.gff, genomic_Amel_Hav3.1_allChrScaf.fna.2.7.7.80.10.50.500.mask: 2-18-2022

GFF3: all lines have correct number of fields
GFF3: no white space problems in fields
GFF3: no type errors with gene/exon
GFF3: all lines have numeric start/stop coordinates
GFF3: all lines have correct strand designations
GFF3: number of genes: 12332
GFF3: number of exons: 267964

GENE DATA
original: ID=gene-LOC551580;Dbxref=BEEBASE:GB42195,GeneID:551580;Name=LOC551580;gbkey=Gene;gene=LOC551580;gene_biotype=protein_coding
name/id: gene-LOC551580
parsed: NC_037638.1 9273 12174 - gene-LOC551580

original: ID=gene-LOC551555;Dbxref=BEEBASE:GB42138,GeneID:551555;Name=LOC551555;gbkey=Gene;gene=LOC551555;gene_biotype=protein_coding
name/id: gene-LOC551555
parsed: NC_037638.1 10792 17180 + gene-LOC551555

original: ID=gene-LOC726347;Dbxref=BEEBASE:GB42194,GeneID:726347;Name=LOC726347;gbkey=Gene;gene=LOC726347;gene_biotype=protein_coding
name/id: gene-LOC726347
parsed: NC_037638.1 17090 23457 - gene-LOC726347

original: ID=gene-Rfwd3;Dbxref=BEEBASE:GB42139,GeneID:409940;Name=Rfwd3;description=ring finger and WD repeat domain 3;gbkey=Gene;gene=Rfwd3;gene_biotype=protein_coding
name/id: gene-Rfwd3
parsed: NC_037638.1 23613 26208 + gene-Rfwd3

original: ID=gene-LOC726145;Dbxref=BEEBASE:GB42193,GeneID:726145;Name=LOC726145;gbkey=Gene;gene=LOC726145;gene_biotype=protein_coding
name/id: gene-LOC726145
parsed: NC_037638.1 28123 30958 - gene-LOC726145

EXON DATA
original: ID=exon-XR_001705491.2-1;Parent=rna-XR_001705491.2;Dbxref=GeneID:551580,Genbank:XR_001705491.2,BEEBASE:GB42195;gbkey=misc_RNA;gene=LOC551580;product=ubiquitin-related modifier 1%2C transcript variant X3;transcript_id=XR_001705491.2
name/id: exon-XR_001705491.2-1
parsed: NC_037638.1 11812 12174 - exon-XR_001705491.2-1

original: ID=exon-XR_001705491.2-2;Parent=rna-XR_001705491.2;Dbxref=GeneID:551580,Genbank:XR_001705491.2,BEEBASE:GB42195;gbkey=misc_RNA;gene=LOC551580;product=ubiquitin-related modifier 1%2C transcript variant X3;transcript_id=XR_001705491.2
name/id: exon-XR_001705491.2-2
parsed: NC_037638.1 11054 11121 - exon-XR_001705491.2-2

original: ID=exon-XR_001705491.2-3;Parent=rna-XR_001705491.2;Dbxref=GeneID:551580,Genbank:XR_001705491.2,BEEBASE:GB42195;gbkey=misc_RNA;gene=LOC551580;product=ubiquitin-related modifier 1%2C transcript variant X3;transcript_id=XR_001705491.2
name/id: exon-XR_001705491.2-3
parsed: NC_037638.1 10913 10994 - exon-XR_001705491.2-3

original: ID=exon-XR_001705491.2-4;Parent=rna-XR_001705491.2;Dbxref=GeneID:551580,Genbank:XR_001705491.2,BEEBASE:GB42195;gbkey=misc_RNA;gene=LOC551580;product=ubiquitin-related modifier 1%2C transcript variant X3;transcript_id=XR_001705491.2
name/id: exon-XR_001705491.2-4
parsed: NC_037638.1 9779 9827 - exon-XR_001705491.2-4

original: ID=exon-XR_001705491.2-5;Parent=rna-XR_001705491.2;Dbxref=GeneID:551580,Genbank:XR_001705491.2,BEEBASE:GB42195;gbkey=misc_RNA;gene=LOC551580;product=ubiquitin-related modifier 1%2C transcript variant X3;transcript_id=XR_001705491.2
name/id: exon-XR_001705491.2-5
parsed: NC_037638.1 9274 9546 - exon-XR_001705491.2-5

FASTA: all sequences have proper characters

There are 114 seqids not in the GFF file (use the '-p gff' option to output)
All seqids in GFF are also in FASTA
See below for list of valid seqids and lengths

Intergenic distance information

Average gene length is 15012.53 bp
Average (pseudo) intergenic distance is 5863.94 bp (max is 554484 bp)
There are 3351 pairs with negative distance (omitted for average)
total genes: 12332 (8981 used for avg distance)
total chr: 61

ANNOTATION 'TYPE' DATA FROM THIS GFF FILE:
CDS
cDNA_match
exon
gene
guide_RNA
lnc_RNA
mRNA
miRNA

primary_transcript
pseudogene
rRNA
region
sequence_feature
snRNA
snoRNA
tRNA
transcript

SEQUENCE-REGIONS WITH GENES/EXONS (bp) -- does not assess other types:

NC_001566.1	16343
NC_037638.1	27754200
NC_037639.1	16089512
NC_037640.1	13619445
NC_037641.1	13404451
NC_037642.1	13896941
NC_037643.1	17789102
NC_037644.1	14198698
NC_037645.1	12717210
NC_037646.1	12354651
NC_037647.1	12360052
NC_037648.1	16352600
NC_037649.1	11514234
NC_037650.1	11279722
NC_037651.1	10670842
NC_037652.1	9534514
NC_037653.1	7238532
NW_020555792.1	22163
NW_020555794.1	40528
NW_020555797.1	41529
NW_020555801.1	31353
NW_020555804.1	21070
NW_020555807.1	17199
NW_020555812.1	6719
NW_020555813.1	4248
NW_020555814.1	95318
NW_020555815.1	67913
NW_020555816.1	40431
NW_020555821.1	35182
NW_020555822.1	24609
NW_020555824.1	13259
NW_020555841.1	73645
NW_020555842.1	60267
NW_020555843.1	44578
NW_020555846.1	23749
NW_020555850.1	18311
NW_020555851.1	10530
NW_020555853.1	5948
NW_020555855.1	4502
NW_020555859.1	486754

NW_020555860.1	311923
NW_020555862.1	120686
NW_020555863.1	108711
NW_020555864.1	92711
NW_020555866.1	66194
NW_020555867.1	56971
NW_020555868.1	56225
NW_020555869.1	52709
NW_020555870.1	52744
NW_020555880.1	30149
NW_020555886.1	25083
NW_020555887.1	24917
NW_020555889.1	22456
NW_020555891.1	21830
NW_020555894.1	21197
NW_020555896.1	19254
NW_020555901.1	15874
NW_020555902.1	15501
NW_020555904.1	14627
NW_020555907.1	13874
NW_020555912.1	12477
NW_020555924.1	9093
NW_020555938.1	5439

There are 114 sequence-regions with no genes/exons (largest is 135562 bp, average 0.00 bp)
