# Supplementary material for: SCRMshaw: Supervised cis-regulatory module prediction for insect genomes
Source: PLoS One. 2024 Dec 5;19(12):e0311752. doi: 10.1371/journal.pone.0311752 (PMC11620701; doi:10.1371/journal.pone.0311752)
Supplement: Attachment — Submitted filename: Response to Review.docx [file pone.0311752.s004.docx]

Response to Review:

We thank the Reviewer and the Editor for their comments on our manuscript. We have made the following changes in response:

1. We added significant new text in the Introduction discussing the general issues surrounding regulatory element discovery, and referenced several review articles that go into detail about various experimental and computational discovery methods. Given that this is a Protocols paper and that we and others have reviewed these methods previously, we did not think it desirable to provide an extensive treatment here. However, the text and cited articles will now allow the interested reader to easily discover more detailed information.

2. Benchmarking regulatory element discovery algorithms is tricky for a large number of reasons, including lack of any true “truth” data sets to benchmark against, and frequently different requirements for input and/or training for the leading approaches. We comment on some of these issues in our earlier paper Asma and Halfon 2019 (PMID: 30953451), which we now cite on line 95. Previous papers on SCRMshaw, which are cited here, do have some benchmarking to the extent possible. Importantly, no good comparisons exist for attempting cross-species discovery, i.e., training on one species and conducting predictions on another, which is the main focus and strength of SCRMshaw. We have added comments on this at lines 93-99 and point out that based on published results “success rates from SCRMshaw appear to be on a par with or better than those from other rigorously-evaluated methods.”

3. We added additional text about SCRMshaw’s limitations at lines 211-218. We also provide a clear reminder that “SCRMshaw predictions are, ultimately, predictions, and appropriate validation experiments should be undertaken for any sequences of particular interest.”

4. With respect to the Editor’s comment “Line 108 [now line 136]: Is this the best heading for this section? ‘Expected Results’”, we followed the template provided by PLoS One for “Protocols” papers, which specifies the section headings. We would be happy to substitute this for a different heading at the Editor’s suggestion.
